# Investigating the prognostic utility of *GSTP1* promoter methylation in prostate cancer

**DOI:** 10.1002/bco2.445

**Published:** 2024-10-30

**Authors:** Ruth Pidsley, Dilys Lam, Wenjia Qu, Phillip Stricker, James G. Kench, Lisa G. Horvath, Susan J. Clark

**Affiliations:** ^1^ Garvan Institute of Medical Research Sydney New South Wales Australia; ^2^ School of Clinical Medicine Faculty of Medicine and Health, UNSW Sydney New South Wales Australia; ^3^ Department of Urology St. Vincent's Sydney New South Wales Australia; ^4^ St Vincent's Prostate Cancer Research Centre Sydney New South Wales Australia; ^5^ Department of Tissue Pathology, NSW Health Pathology Royal Prince Alfred Hospital Sydney New South Wales Australia; ^6^ Chris O'Brien Lifehouse, Missenden Road Camperdown New South Wales Australia; ^7^ University of Sydney Sydney New South Wales Australia; ^8^ Present address: School of Molecular Sciences The University of Western Australia Crawley Western Australia Australia; ^9^ Present address: Harry Perkins Institute of Medical Research Nedlands Western Australia Australia

**Keywords:** biomarker, diagnostic, DNA methylation, epigenetics, *GSTP1*, prognostic, prostate cancer

## Abstract

**Objectives:**

We aim to determine the prognostic significance of *glutathione S‐transferase Pi 1* DNA methylation (*mGSTP1*) in two independent prostate cancer cohorts with long‐term clinical follow‐up data.

**Subjects/Patients and Methods:**

We first re‐examined a published, in‐house whole genome bisulphite sequencing (WGBS) prostate cancer dataset, derived from radical prostatectomy (RP) tissue (*n* = 15) with median follow‐up 19.5 years, to confirm and visualise the association between *mGSTP1* and patient mortality. To validate prognostic significance, we used a quantitative methylation‐specific head‐loop (MS‐HL) assay to measure *mGSTP1* levels in a larger, independent cohort (*n* = 186), and performed univariable and multivariable Cox survival analysis.

**Results:**

Re‐analysis of WGBS data showed a significant increase in *mGSTP1* in RP samples from patients with lethal versus non‐lethal disease. Subsequent analysis in the larger cohort using the MS‐HL assay confirmed that *mGSTP1* was detectable in 97% of RP samples, validating the diagnostic potential of *mGSTP1*. Univariable Cox survival analysis revealed a significant association between *mGSTP1* levels and biochemical recurrence and metastatic relapse free survival, with a near‐significant association with prostate cancer specific mortality. Notably, multivariable Cox models demonstrated that *mGSTP1* did not add independent prognostic value beyond standard clinicopathological features.

**Conclusion:**

Our study supports the importance of DNA methylation as a tissue‐based prostate tumour biomarker. *GSTP1* methylation is well established as a diagnostic marker, and in this study, we find that *GSTP1* methylation levels are also associated with disease prognosis. Further research is required into the clinical utility of prognostic methylation markers and their functional role in disease progression.

## 1 INTRODUCTION

Prostate cancer is the most common non‐cutaneous cancer in men. In 2020 alone, over 1.4 million new prostate cancer cases were diagnosed and >375 000 related deaths reported worldwide.^1^ The majority of men are diagnosed with disease localised to the prostate gland^2^ and, if treatment is recommended, will commonly have the whole prostate gland surgically removed via a procedure termed radical prostatectomy (RP). Whilst curative for many, a large proportion (~40%) of patients undergoing RP will experience a biochemical recurrence (BCR) which is a rise in prostate specific antigen (PSA) levels in the blood indicating the recurrence of the tumour.^3^ Critically, 5%–10% of these patients will progress to lethal‐metastatic disease.^3^ Treatment decisions take into account clinicopathological measures including PSA levels, International Society of Urological Pathologists (ISUP) Grade Group pathological score, pathological T‐category and surgical margin status. However, these clinical prognostic factors lack accuracy for patient risk‐stratification.^4^ Improved prognostic biomarkers are needed to identify the patients most at risk of recurrence to help guide clinician decision making and improve patient outcomes.

There is increasing interest in the use of molecular biomarkers for disease prognosis.^5^ DNA methylation is particularly advantageous as a molecular biomarker as it is a stable and heritable DNA modification and is a recognised hallmark of cancer.^6^ Indeed, in our recent study, we identified novel genomic regions at which DNA methylation levels were associated with prostate cancer specific mortality (PCSM) and improved PCSM prediction relative to standard clinical factors alone.^7^

The most widely studied methylation biomarker in prostate cancer research to date is the *Glutathione S‐transferase Pi 1* (*GSTP1*) gene CpG island promoter region.^8^ *GSTP1* is well‐characterised as a detoxifying enzyme and tumour suppressor, and aberrant methylation of the *GSTP1* promoter has been associated with tumour development and prognosis in a range of tumour types.^9^, ^10^ The CpG island promoter region of *GSTP1* is typically unmethylated in normal prostate tissue DNA but largely hypermethylated in prostate tumour tissue DNA.^11^ Data from The Cancer Genome Atlas Prostate Adenocarcinoma (TCGA PRAD) cohort showed that *GSTP1* was methylated in >85% of tumour samples, with concomitant suppression of *GSTP1* mRNA expression.^12^ The ubiquity of *GSTP1* promoter methylation (*mGSTP1)* in prostate cancer, throughout all stages of disease progression, suggests that it is an early, clonal event in tumourigenesis.^9^

The high specificity of *GSTP1* promoter methylation to tumour tissue makes it an ideal diagnostic biomarker.^13^ Indeed, ConfirmMDx (MDxHealth, Irvine, CA, USA), the first commercially available diagnostic methylation test for prostate cancer,^14^ uses a quantitative methylation specific PCR (qMSP) to measure promoter methylation at *GSTP1* (and two additional genes, *APC* and *RASSF1*) to detect cancer in histologically negative biopsies.^15^ However, the prognostic value of *mGSTP1* levels in prostate tumour tissue is less clear.^5^ A number of studies have investigated the association between the levels of *mGSTP1* in tissue and prostate cancer survival, with mixed conclusions (reviewed in Lam et al.^5^). The lack of consistent results is likely due to factors including differences in the methylation assays used; clinical features and size of each cohort; clinical follow‐up period; and survival endpoints used in analysis. For example, many studies use BCR as a survival endpoint; however, unlike metastatic relapse (MR), it is not a good surrogate for PCSM.^16^

Given the suggestive but inconsistent results of studies exploring the prognostic potential of *mGSTP1* in prostate cancer we chose to conduct our own investigation. We elected to use a quantitative and sensitive methylation‐specific head‐loop (MS‐HL) assay for measuring *mGSTP1* levels, previously developed in our laboratory.^13^, ^17^, ^18^ Importantly, we used patient cohorts with long‐term follow up data (median: 15 years), required for the clinically relevant end‐points of MR and PCSM to occur^19^ and analysed each survival endpoint separately.

## 2 MATERIALS/SUBJECTS AND METHODS

### 2.1 *GSTP1* methylation whole genome bisulphite sequencing data

We utilised a published prostate cancer methylome dataset from our laboratory,^7^ for re‐evaluation and visualisation of Whole Genome Bisulphite Sequencing (WGBS) data, derived from RP tissue of patients with lethal disease (*n* = 7) and non‐lethal (*n* = 8) disease (median follow‐up 19.5 years). The WGBS data corresponds to BigTable.tsv.gz in NCBI GEO repository GSE158927; a tsv file providing coverage and methylation data for each sample (columns) at each CpG site (rows). See Table S1a for details of the clinical cohort. Methylation at the *GSTP1* CpG island differentially methylated region (DMR) was visualised using the *DMR.plot* function in DMRcate.^20^

### 2.2 Study population

The study cohort comprised 186 prostate cancer patients selected with ISUP Grade Group 2 or greater, who underwent Radical Prostatectomy (RP) treatment between 1997 and 2003 at the St Vincent's Hospital^7^, ^16^, ^21^ (see Table S1b for details of the clinical cohort). Over an extensive follow‐up period (median: 15 years, range: 0.8–22 years), 86 patients experienced BCR, 25 patients had MR, and 16 patients died of prostate cancer. One patient was removed from the study due to missing pre‐operative PSA data, leaving *n* = 185 patients.

### 2.3 Tumour tissue preparation, DNA extraction and bisulphite‐conversion

Samples were processed as described in Pidsley and colleagues.^7^ Briefly, archival formalin‐fixed paraffin‐embedded (FFPE) prostate tumour tissue blocks were obtained from RP specimens. Haematoxylin and eosin staining on prostate tissue specimens were reviewed by a uropathologist (J.K.) to mark and confirm the presence and location of prostate cancer tumour areas. For each patient, five 1 mm tumour tissue cores from within histologically verified tumour region (at least 50% neoplastic cells [typically >70%]) were taken for genomic DNA extraction. DNA was extracted using the AllPrep FFPE DNA/RNA Kit (Qiagen, Cat. No. 80234), quantified with the Qubit dsDNA HS Assay Kit (Life Technologies, USA) and stored at −80°C until use. Extracted DNA was bisulphite treated using the EZ DNA Methylation‐Lightning Kit (Zymo Research, USA, Cat. No. D5030 & D5033) according to the manufacturer's instructions.

### 2.4 Methylation‐specific head‐loop (MS‐HL) PCR *GSTP1* assay

The methylation‐specific head‐loop (MS‐HL) PCR assay was performed as previously described to measure the absolute amount of *mGSTP1* DNA in the samples.^17^ Briefly, following bisulphite treatment of the study cohort samples, a *GSTP1* CpG island‐specific head‐loop primer was used that causes looping back and extension on sequences derived from DNA not methylated at CpG sites, to selectively suppress amplification of unmethylated sequences.

Forward primer: 5′ACACAACCCACATCCCCAAAATGTTGGGAGTTTTGAGTTTTATTTT; Reverse primer: 5′AAAACCICIAAACCTTCICTAAAATTTC; Probe: 5′VIC‐TCG CCG CCG CAA T‐MGBNFQ. A control *GSTP1* PCR reaction was used to ensure that the locus had not been somatically deleted in the cohort tumour samples. Forward primer: 5′GGGATTATTTTTATAAGGTTYGGAGGT; Reverse primer: 5′AAAACCCRAACCTAATACTACRAATTAA. Sybr green was used to quantify DNA. PCR reaction conditions: 95°C for 120 s, 60 cycles at 95°C for 15 s, 60°C for 60 s. Real‐time PCR was carried out in triplicate using an ABI PRISM® ABI7900 Sequence Detection System. The average triplicate Ct values from the *GSTP1* MS‐HL PCR assay were used to estimate the quantity of *mGSTP1* using a standard curve, generated using known concentrations of SssI fully methylated DNA (1, 5, 10, 50, 100 and 500 ng), as described previously.^17^ Detectable *mGSTP1* was defined as >1 ng sample.

### 2.5 Statistical analysis

All statistical analyses were conducted using R (version ≥3.2.2). Chi‐square tests and Wilcoxon rank sum tests were used to assess the association between *mGSTP1* and known prognostic clinicopathological factors, assessed as dichotomous variables: ISUP Grade Groups (2 vs. ≥3), pathological T‐category (≤pT2 vs. ≥pT3), pre‐op PSA levels (<10 ng/mL vs ≥ 10 ng/mL) and surgical margin status (negative vs. positive). *mGSTP1* was assessed as a dichotomous variable with patients divided into high or low methylation groups, based on the methylation value at the 75th percentile cut‐off.

The prognostic value of *mGSTP1* was tested using survival outcomes: BCR, defined as a serum PSA concentration ≥0.2 ng/mL increasing over a 3‐month period; MR, determined by biopsy or positive scan(s) confirming local, visceral or bony metastasis; and PCSM, with deaths identified from the NSW State Cancer Registry and cause of death confirmed by contacting patients' GP and through review of medical records. For survival analysis, Kaplan–Meier, log‐rank tests and univariable and multivariable Cox proportional hazard models were performed, with significance set at *p* < 0.05. For multivariable Cox analyses, the statistically equivalent signatures (SES) feature selection algorithm, part of the ‘MXM’ R package, was used to identify minimal‐size predictive signatures with maximal predictive power by performing a variant of forward selection.^22^ Using the inbuilt conditional independence test for survival analysis (Cox regression, ‘censIndCR’ test), the input variables were ISUP Grade Group, pathological T‐category, PSA level, surgical margin status and *mGSTP1*. Harrell's concordance index was used to measure the predictive discrimination of the multivariable Cox proportional hazard models for the three survival outcomes.^23^

### 2.6 Ethics approval and consent to participate

All tissue samples used were from the Garvan Institute/St Vincent's Prostate Cancer biobank with written informed consent and human ethics approval (SVH File Number 12/231).

## 3 RESULTS

### 3.1 Whole genome bisulphite sequencing data from primary prostate cancer samples reveals a difference in *mGSTP1* levels between patients with non‐lethal and lethal disease

**FIGURE 1:**
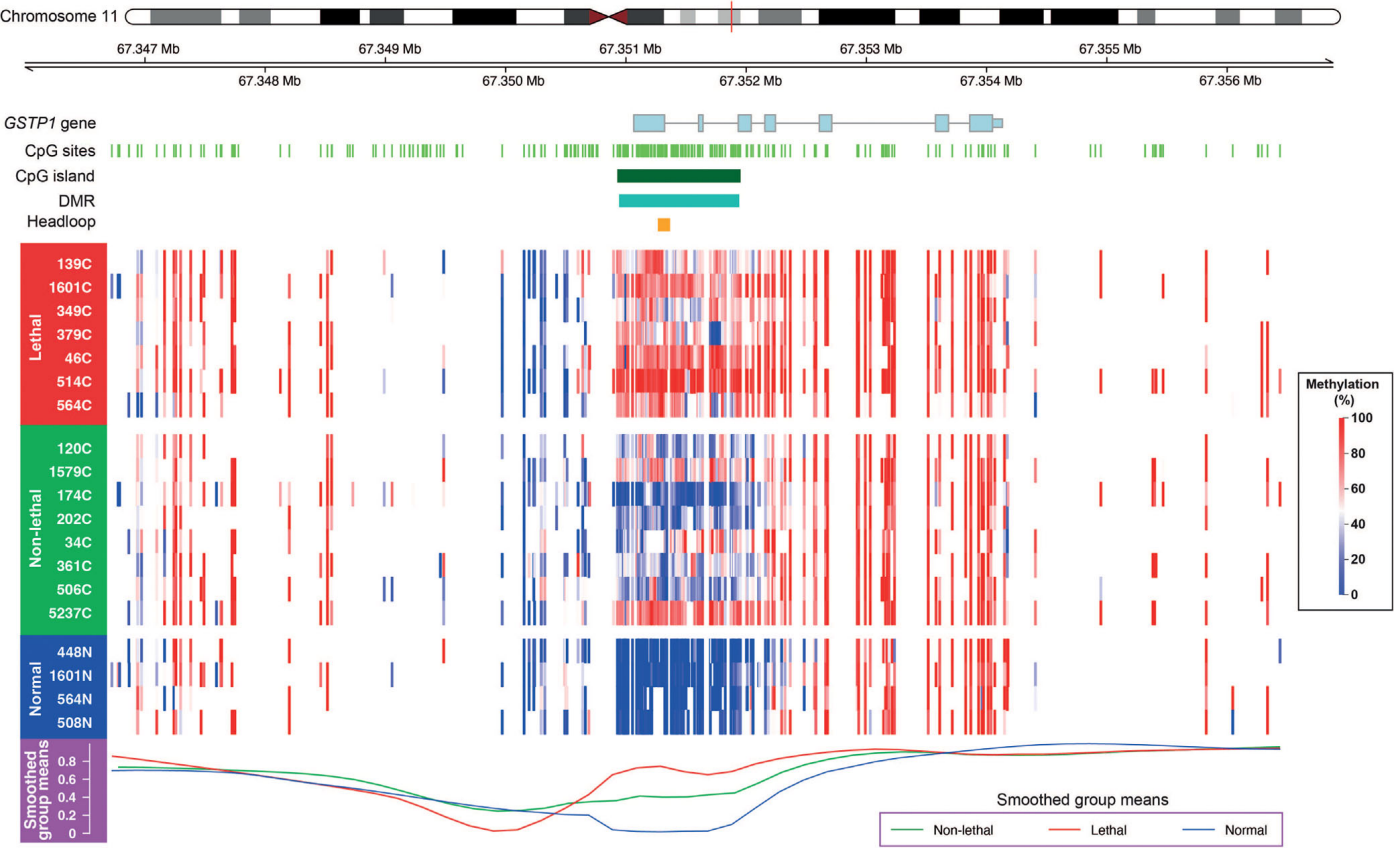
Increased methylation at the *GSTP1* promoter CpG island in tumour tissue of patients with lethal prostate cancer compared to non‐lethal prostate cancer. DMRcate heatmap of *GSTP1* DMR showing methylation across tumour tissue samples from individual patients in the non‐lethal (green) versus lethal (red) groups, and normal adjacent tissue (blue). Smoothed group means are shown in a line plot at the bottom. The *GSTP1* gene is represented by grey lines, with light blue rectangles representing exons, the promoter CpG island is represented by a dark green bar and the head‐loop target region by a dark yellow rectangle.

To investigate whether DNA methylation levels at the *GSTP1* promoter region are associated with prostate cancer survival, we first re‐examined a published, in‐house whole genome bisulphite sequencing (WGBS) dataset derived from FFPE RP samples.^7^ The dataset was from an archival cohort of 15 patients with localised prostate cancer, who had undergone RP, including *n* = 8 patients with non‐lethal disease (alive > 10 years post RP) and *n* = 7 patients with lethal disease (died of prostate cancer ≤ 10 years post RP) as well as 4 normal prostate tissue samples (see Table S1a for cohort details). Using the same cohort we previously reported a list of 1420 differentially methylated regions (DMRs) between the lethal and non‐lethal groups. On re‐examination we found that the 711th most significant hypermethylated DMR (chr11:67350944‐67 351 942, hg19) from this list was located at the *GSTP1* promoter, encompassing 61 CpG sites. The mean CpG methylation across the DMR for each group was lethal = 69.52%, non‐lethal 41.50% and adjacent normal tissue = 3.77%, with minimum adjusted *p*‐value 7.65e‐20 between the lethal and non‐lethal groups. Visualisation of the region (Figure 1) shows that the DMR spans the entire *GSTP1* promoter CpG island and almost all 61 CpG sites are unmethylated in the adjacent normal tissue. The majority of tumour samples exhibit DNA hypermethylation across the region, but notably the non‐lethal group show more within‐ and between‐sample heterogeneity than the lethal group samples.

### 3.2 Association between *GSTP1* promoter methylation and patient survival using a quantitative methylation‐specific head‐loop (MS‐HL) assay

To further validate an association between *mGSTP1* levels and patient survival we used the methylation‐specific head‐loop (MS‐HL) assay to quantify *mGSTP1* DNA levels in FFPE tumour tissue from a well‐characterised, population‐based cohort of 185 patients with localised prostate cancer, with median 15 years follow‐up and ISUP Grade Group ≥ 2^21^ (see Table S1b for cohort details). Figure 1 shows the 101 bp region (chr11:67351266‐67 351 367, hg19) that is assessed by the *GSTP1* MS‐HL assay^17^ and sites within the reported *GSTP1* DMR.

**FIGURE 2:**
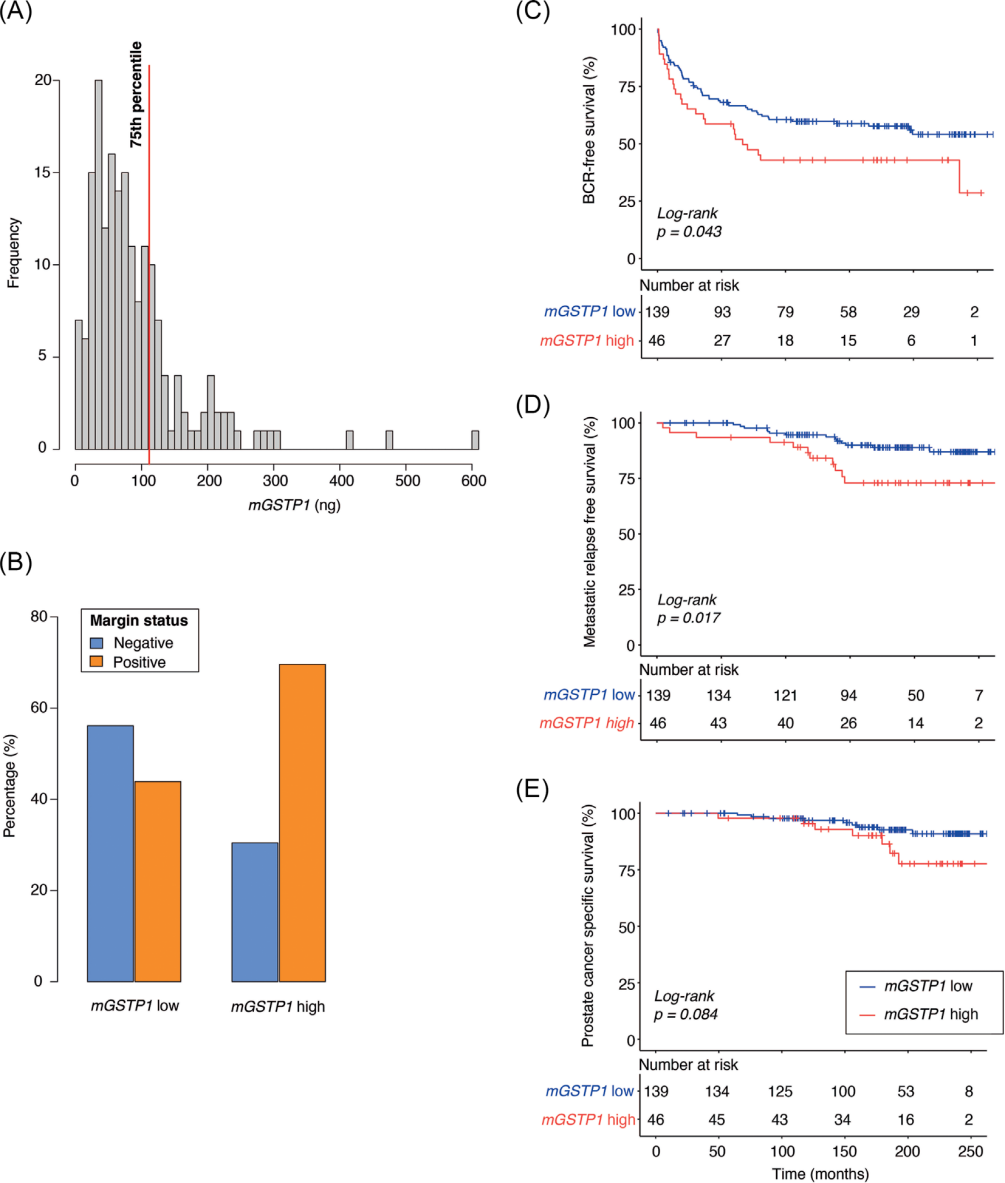
MS‐HL *mGSTP1* levels associated with patient survival. (A) Histogram of *mGSTP1* levels of *n* = 185 patient prostate tumour samples, showing dichotamisation threshold for *mGSTP1* low (≤75th percentile) and *mGSTP1* high (>75th percentile), (B) Bar chart of association between dichotamised *mGSTP1* and margin status. Prognostic potential of *mGSTP1*: Kaplan–Meier survival curves with endpoints of (C) biochemical recurrence, (D) metastatic relapse and (E) prostate cancer‐specific mortality. Red line indicates *mGSTP1* high and blue line indicates *mGSTP1* low.

Using the MS‐HL assay, we found that *mGSTP1* was detectable in 180 out of the 185 tumour samples (97%). For the five samples with undetectable methylation the *mGSTP1* the value was set to 0 ng. *mGSTP1* levels ranged from 0 to 601.85 ng DNA, with median: 72.74 ng and 75th percentile: 111.63 ng. Based on the approach taken in our previous study^7^ we used the 75th percentile (111.63 ng) as a threshold to binarise the data into ‘*mGSTP1* high’ and ‘*mGSTP1* low’ (Figure 2a). Wilcox and chi‐square tests were performed to evaluate the association between binarised *mGSTP1* and individual clinicopathological variables: pre‐operative PSA (ng/mL), Pathological T‐Category, ISUP Grade Group and Margin status (Table 1). Only Margin status (negative vs. positive) was significantly associated with *mGSTP1* levels, showing ‘*mGSTP1* high’ was more common in margin positive cases than margin negative cases (Figure 2b).

**TABLE 1:** Wilcoxon rank sum test and chi‐square tests of *mGSTP1* (ng) and clinicopathological features.

Variable	Statistical test	Dichotomisation thresholds	Statistic	*p*‐value
Continuous				
Pre‐operative PSA (continuous)	Wilcoxon rank sum test	NA	2687.5	0.11
Dichotomised				
Pre‐operative PSA	Chi‐square test	<10 vs. ≥10 ng/mL	1.18	0.28
Pathological T‐category	Chi‐square test	pT2 vs. ≥pT3	0.02	0.90
Pathological ISUP grade group	Chi‐square test	2 vs. 3–5	0.001	0.97
Margin status	Chi‐square test	Negative vs. positive	8.12	**4.38E‐03**

*Note*: *GSTP1* methylation levels binarised using 75th percentile cut‐off. Values in bold indicate statistically significant at *p* < 0.05.

Next, log‐rank and univariable Cox regression analysis were used to assess the association between binarised *mGSTP1* and three clinical end‐points: BCR, MR and PCSM. In the log‐rank tests, *mGSTP1* was associated with BCR‐free survival (*p* = 0.043) and MR‐free survival (*p* = 0.017), with a near‐significant association with PCSM (*p* = 0.084) (Table 2). In all cases ‘*mGSTP1* high’ was associated with poorer survival. Similar results were observed in the Cox regression analysis [BCR: *p* = 0.045, HR = 1.59 (1.01–2.51), Figure 2c; MR: *p* = 0.021, HR = 2.54 (1.15–5.59), Figure 2d; PCSM: *p* = 0.093, HR = 2.33 (0.87–6.27), Figure 2e] (Table 2). To compare the predictive power of *mGSTP1* levels with established prognostic markers, we performed forward selection with multivariable Cox regression using predictive variables *mGSTP1* and the four clinicopathological factors. The final multivariable prognostic models for each of the three clinical endpoints are summarised in Table S2. *mGSTP1* was not selected in the final models for any of the input variable combinations at any clinical end‐points.

**TABLE 2:** Log‐rank tests and univariable cox regression analyses of clinicopathological features and *mGSTP1* (ng) in the *GSTP1* head‐loop cohort (*n* = 185).

Variable	Dichotomisation thresholds	Biochemical recurrence (BCR)			Metastatic relapse (MR)			Prostate cancer mortality (PCSM)		
		Log‐rank	Cox regression		Log‐rank	Cox regression		Log‐rank	Cox regression	
		*p*‐value	HR (95% CI)	*p*‐value	*p*‐value	HR (95% CI)	*p*‐value	*p*‐value	HR (95% CI)	*p*‐value
DNA methylation										
*mGSTP1* (ng)	<75th vs. ≥75th percentile	**0.043**	1.59 (1.01–2.51)	**0.045**	**0.017**	2.54 (1.15–5.59)	**0.021**	0.084	2.33 (0.87–6.27)	0.093
Clinicopathological features										
Pre‐operative PSA	<10 vs. ≥10 ng/mL	**0.001**	2.05 (1.34–3.14)	**0.001**	**0.026**	2.39 (1.08–5.26)	**0.031**	0.074	2.39 (0.89–6.44)	0.083
Pathological T‐category	pT2 vs. ≥pT3	**0.004**	1.88 (1.21–2.92)	**0.005**	**0.019**	2.74 (1.14–6.56)	**0.024**	0.120	2.28 (0.79–6.55)	0.128
Pathological ISUP grade group	2 vs. 3–5	**0.000**	2.26 (1.48–3.46)	**0.000**	**0.000**	5.88 (2.35–14.72)	**0.000**	**0.002**	4.99 (1.61–15.49)	**0.005**
Margin status	Negative vs. positive	**0.001**	2.08 (1.35–3.23)	**0.001**	**0.008**	3.07 (1.28–7.36)	**0.012**	**0.024**	3.40 (1.09–10.54)	**0.034**

*Note*: Values in bold indicate statistically significant at *p* < 0.05.

## 4 DISCUSSION

This study shows that CpG island *GSTP1* methylation levels in tumour DNA at the time of RP is associated with patient prognosis in two independent prostate cancer cohorts with long‐term follow‐up clinical data (median: >15 years), but is outperformed by standard clinicopathological features in multivariable models of prostate cancer survival.

Re‐examination of our previously published methylome data from our WGBS cohort showed that *mGSTP1* was associated with patient survival, with a significant and sizable methylation difference between the lethal and non‐lethal patient groups.^7^ *GSTP1* was not selected as a candidate region for validation in the prior study due to its relatively low ranking, however given the ~30% methylation difference between patient groups, and its association with disease recurrence in the literature,^24^, ^25^ validation in an independent larger patient cohort was warranted. In this second cohort of prostate cancer patients, we used a quantitative MS‐HL assay to measure *GSTP1* methylation and perform survival analysis with the endpoints BCR, MR and PCSM. Log‐rank and univariable Cox regression confirmed that *mGSTP1* was associated with BCR and MR, with a trend towards a significant association with PCSM. However, in multivariable Cox models including clinicopathological variables, *mGSTP1* was not selected as a significant independent prognostic variable.

A variable will only be selected in a multivariable model if it adds independent information, which will not be the case if it is strongly correlated with other variables in the model. Consistent with this, our results showed that *mGSTP1* was significantly associated with margin status, and margin status was selected in the final multivariable model for all three survival endpoints. The lack of *mGSTP1* selection in the final model is in agreement with the large (*n* = 367) study by Vasiljevic and colleagues,^25^ in which prostate tumour *GSTP1* methylation levels were associated with PCSM in univariable Cox analysis but eliminated in selection for the final multivariable Cox model. Likewise, another large study (*n* = 452) by Maldonado and colleagues^24^ found association between prostate tumour *GSTP1* methylation levels and prostate cancer recurrence in univariable analysis, but not after adjustment for multiple clinical variables. Only in a secondary analysis, in which they split the cohort into early‐ and late‐stage disease, did they find an association after adjustment for clinical variables between increased *GSTP1* methylation and a higher risk of recurrence in early‐stage disease only. The Maldonado and colleagues cohort included patients with Gleason 6 (ISUP grade 1) (~15%) whilst our cohort only included patients with ISUP grade 2 or above. Maldonado and colleagues also performed chi‐square tests, instead of Cox models which include survival times, and defined ‘recurrent cases’ as patients with *any* of BCR, clinical recurrence (local recurrence), systemic metastasis and/or PCSM.^24^ Thus, differences in the clinical characteristics of the cohort and different analytical approaches could explain the discrepancies in our results.

The visualisation of methylation at *GSTP1* in the WGBS cohort provides a clear illustration of the reason that *GSTP1* promoter methylation performs so well as a highly specific *diagnostic* marker of prostate cancer (e.g., previous works^15^, ^26^): the locus is almost entirely unmethylated in normal tissue, and methylated in all tumour tissue, at a range of levels. Consistent with this we found that in the *GSTP1* head‐loop patient cohort (*n* = 185), 97% of tumour samples had detectable levels of *mGSTP1* as measured by MS‐HL *GSTP1* assay. Together these results support the concept that *GSTP1* methylation is one of the earliest and most common molecular events in prostate cancer, likely due to clonal expansion.^9^, ^27^ Therefore, *mGSTP1* levels may simply represent the load of tumour epithelial cells within each prostate tissue sample. Indeed, investigations in TCGA PRAD data show correlation between *GSTP1* methylation and pathology measures of tumour purity.^28^ We have also shown previously that *mGSTP1* is highly specific to tumour epithelial cells, whereas normal prostate epithelial cells and prostate stromal cells show complete hypomethylation.^29^

A *mGSTP1* measure representing tumour load may still have prognostic value clinically, for example, it could provide an indication of tumour volume, which is itself associated with disease risk.^26^, ^30^ Assessment of *mGSTP1* as a proxy for tumour load in biopsy cores targeted to regions of the prostate where positive margins commonly occur (apex or base) could also provide *pre‐operative* prognostication of margin status, itself known to be a highly prognostic clinical variable of BCR in prostate cancer.^31^ Indeed, the association between *mGSTP1* in RP tumour tissue specimens and positive margin status in this current study supports the feasibility of this application. Furthermore, the high specificity of *mGSTP1* to prostate tumour tissue makes it an ideal biomarker for monitoring tumour load in the bloodstream for detection of metastasis, treatment response or disease recurrence after treatment.^13^, ^18^, ^32^, ^33^

In summary, our current data support DNA methylation as a tissue‐based prostate tumour biomarker. Even though *mGSTP1* is well‐established as a diagnostic marker it may potentially provide a prognostic biomarker in specific clinical contexts as discussed. However, in terms of biopsy or RP tissue‐based tests, levels of DNA methylation at other genomic loci are likely to be superior as independent predictors of prostate cancer recurrence. We speculate that, rather than an early and ubiquitous hypermethylation event such as that occurring at the *GSTP1* promoter, methylation changes that gradually accumulate with disease progression or are potentially causative for disease progression will provide more biologically informative prognostic methylation biomarkers. These epigenetic changes could be within the tumour cells or within other cells in the tumour microenvironment.^34^, ^35^ Single cell methylation studies of prostate tumours at different disease stages and Grade Groups are necessary to clarify the role and sequential order of different methylation alterations in tumour progression, to advance the development of prognostic biomarkers and identification of new epigenetic driver mutations of malignancy.

## AUTHOR CONTRIBUTIONS

Ruth Pidsley, Lisa G. Horvath and Susan J. Clark conceived the study. Ruth Pidsley and Susan J. Clark supervised the research. Wenjia Qu and Dilys Lam performed the MS‐HL *GSTP1* PCR assay and sequencing experiments. Ruth Pidsley and Dilys Lam interpreted the data. James G. Kench provided pathology evaluation. Phillip Stricker, James G. Kench and Lisa G. Horvath supplied and curated prostate samples from St Vincent's Prostate Cancer biobank. Ruth Pidsley and Susan J. Clark wrote the manuscript and all authors reviewed the manuscript.

## CONFLICT OF INTEREST STATEMENT

The authors have no conflict of interest to declare.

## Supporting information


**Table S1.** Clinicopathological characteristics of cohorts.
**Table S2.** Results of the multivariable Cox regression analyses of *mGSTP1* in the *mGSTP1* head‐loop cohort (n = 185), showing models with the greatest predictive power selected using the SES feature selection algorithm with *mGSTP1* and clinicopathological features as input variables.

## Data Availability

WGBS data analysed as part of this study is available from NCBI Gene Expression Omnibus (GEO) (www.ncbi.nlm.nih.gov/geo) under accession number GSE158927. The datasets generated during the current study are available from the corresponding author on reasonable request.
